# Knowledge, Attitude, and Practices of Barbers on Hygiene and Prevention of Communicable Diseases

**DOI:** 10.7759/cureus.98144

**Published:** 2025-11-30

**Authors:** Muhammad Bilal Khattak, Suleman Akhter, Fida Muhammad, Asif Rasheed, Amir Moavia, Habiba Qazi, Muhammad Usman Sharif, Bilal Ahmed, Shahkar A Khan, Naveed Ahmed

**Affiliations:** 1 Internal Medicine, Khyber Teaching Hospital, Peshawar, PAK; 2 Trauma and Orthopaedics, Manchester University NHS Foundation Trust, Manchester, GBR; 3 Public Health, Ayub Medical College, Abbottabad, PAK; 4 Public Health, Health Services Academy, Islamabad, PAK; 5 Emergency, Mufti Mehmood Memorial Teaching Hospital, Dera Ismail Khan, PAK; 6 General Medicine, KMU Institute of Medical Sciences Kohat, Kohat, PAK; 7 Pulmonology, Ayub Teaching Hospital, Abbottabad, PAK; 8 Medicine, Ayub Medical College, Abbottabad, PAK; 9 Medicine and Surgery, Ayub Medical College, Abbottabad, PAK; 10 Internal Medicine, Ayub Teaching Hospital, Abbottabad, PAK

**Keywords:** barbers, communicable diseases, hepatitis b, hepatitis c, hiv, hygiene practices, infection control, occupational health, public health

## Abstract

Background: Barbers are frequently exposed to potential infection sources due to the use of sharp instruments, posing a risk for transmission of communicable diseases, such as hepatitis B, hepatitis C, and HIV. Limited awareness and poor hygienic practices among barbers in low- and middle-income countries contribute to the spread of these infections. This study aimed to assess the knowledge, attitudes, and practices of barbers regarding hygiene and communicable diseases in Abbottabad, Pakistan.

Methods: A cross-sectional study was conducted from July to November 2024 among 65 barbers selected through convenience sampling from three areas of Abbottabad-Mandian, Kalapul, and Main Bazar. Data were collected using a structured questionnaire and an observational checklist assessing knowledge, preventive practices, and hygiene standards. Descriptive analysis was performed using Statistical Product and Service Solutions (SPSS, version 27; IBM SPSS Statistics for Windows, Armonk, NY).

Results: Most barbers (75.4%) were aware that diseases could be transmitted through their work, but only 40% correctly identified specific infections, such as hepatitis B, hepatitis C, and HIV. While 58.5% of shops had sterilizers, only 63% of barbers consistently used them. Observational findings also showed 95% used new blades per client, and 94% had antiseptics available. Most barbers (75.4%) were aware that diseases could be transmitted through their work, but only 40% could correctly identify specific infections such as hepatitis and HIV. Dettol chloroxylenol was the most commonly used disinfectant (78.4%), with varying combinations of sterilizers and new blades. Observational findings showed that 95% used new blades per client, 94% had antiseptics available, and 58.5% possessed sterilizers. However, 47% maintained visibly clean environments.

Conclusion: Barbers in Abbottabad demonstrated partial knowledge regarding communicable diseases, with significant gaps in identifying specific infection risks associated with their profession. Although several basic hygiene practices - such as the use of new razor blades - were commonly observed, sterilization and overall infection-control measures remained inconsistent. Strengthening targeted educational initiatives and promoting standardized hygiene practices are essential to improving safe barbering practices and reducing the risk of disease transmission in this setting.

## Introduction

Health has been declared a fundamental human right [1]. Despite this fact, many people worldwide remain deprived of this right due to the heavy burden of disease. Developing countries, in particular, face a double burden of communicable and non-communicable diseases. Communicable diseases, also referred to as infectious diseases, result from the invasion and multiplication of disease-causing agents in body tissues, followed by host tissue reactions to these agents and their toxins [2]. Barbers are at increased occupational risk of acquiring and transmitting communicable diseases due to frequent use of sharp instruments and direct contact with clients’ blood and skin. Such exposures can lead to infections such as hepatitis B, hepatitis C, and HIV, which not only affect the health of barbers themselves but also pose a public health risk. In Pakistan, barbering practices extend beyond hair cutting to services such as scalp massaging, nail trimming, and minor surgical procedures, highlighting the need for proper hygiene and infection control practices [3].

Communicable diseases remain a leading cause of morbidity and mortality in low- and middle-income countries. In Pakistan, there is endemic hepatitis B and C with an estimated 7.6% affected individuals, the fifth highest tuberculosis burden in the world, and an increasing prevalence of HIV [4]. Transmission of such infections is strongly influenced by behavioral and environmental factors, including awareness, motivation, and knowledge of individuals performing high-risk procedures, such as barbers. Lack of hygienic practices and improper sterilization techniques in barbering contribute significantly to the spread of infections, making this occupational group an important public health concern.

Barbers are frequently exposed to skin contact and use of reusable sharp instruments, which pose a risk for transmitting pathogens if proper sterilization and decontamination procedures are not followed. Effective sterilization methods include dry heat, flame, and ultraviolet light, while chemical agents such as alcohol, phenol compounds, iodine, chlorine, and quaternary ammonium compounds are also recommended [5]. Failure to use these consistently can result in the spread of viral infections, such as hepatitis B, hepatitis C, HIV/AIDS, and herpes; bacterial infections such as folliculitis, impetigo, eczema, warts, barber’s itch, and tetanus; and parasitic and fungal infections such as scabies and tinea capitis [6]. In such circumstances, health education plays a vital role in changing attitudes and practices, thereby preventing the spread of these infections.

Several studies from different regions highlight the gaps in knowledge and practices among barbers. In Nigeria, only 72.5% of barbers disinfected their instruments, often using unsafe agents such as kerosene [7]. In Ethiopia, while 99% of barbers were aware of sterilization practices, only 51% recognized the risk of HIV transmission through contaminated instruments [8]. In India, most barbers knew about HIV/AIDS, but only a small proportion realized that their profession carried a direct risk of transmission [9]. In Pakistan, awareness regarding blade sharing as a route of HIV transmission was high among barbers working in larger salons but significantly lower in smaller and roadside barbers, with inconsistent use of new blades and antiseptics between customers [10]. Hepatitis B and C are major causes of chronic liver disease, hepatocellular carcinoma, cirrhosis, and end-stage liver disease. A large-scale study conducted by the Pakistan Health Research Council (2007-2008) found an overall prevalence of 7.6% for hepatitis B and C in Pakistan, with around 13 million people estimated to be infected [11]. In Yemen, 234 barbers were surveyed, and although 73.1% had heard of hepatitis B and C, awareness regarding specific modes of transmission and preventive practices was limited, with poor compliance with hygiene practices [12].

Studies from Nigeria, Ethiopia, India, Pakistan, and Yemen consistently show that, while barbers often have general awareness of disease transmission, their specific knowledge of infections such as hepatitis B, hepatitis C, and HIV is limited, and hygiene practices are applied inconsistently. Common barriers include low education, limited formal training, and socioeconomic constraints. Despite these findings, no comprehensive assessment exists for barbers across Pakistan, where diverse urban and socioeconomic conditions may influence knowledge, attitudes, and practices regarding hygiene and communicable disease transmission. This study addresses this gap by providing a context-specific evaluation of barbers’ awareness of occupational health hazards, attitudes toward preventive measures, and actual hygiene practices, including the use of sterilizers, antiseptics, and maintenance of shop cleanliness. The findings are intended to identify gaps in knowledge and preventive behaviors and to guide targeted health education and policy interventions tailored to this high-risk occupational group.

## Materials and methods

Study area

The study was conducted in Abbottabad, Pakistan. Abbottabad city was divided into three urban subdivisions - Mandian, Kalapul, and Main Bazar - based on a geographical gradient extending from the city entrance to its central commercial zone. These areas were selected due to their high density of barber shops and representation of different socioeconomic zones. Barbers were selected using a convenience sampling method, and a total of 65 barbers were included across these divisions, representing both shop owners and hired workers.

Study design and population

A descriptive cross-sectional study design was employed to assess the knowledge, attitudes, and practices of barbers regarding hygiene and communicable disease transmission in Abbottabad. The study population included all consenting barbers working within the selected divisions during the data collection period.

Sampling technique

Barbers were recruited using convenience sampling across the three divisions. Before recruitment, all barber shops located along the main commercial streets and adjoining lanes of the selected areas were mapped, identifying approximately 120-130 shops in Mandian, Kalapul, and Main Bazar. Shops that were open during field visits were approached sequentially during business hours. All barbers aged ≥16 years, working for at least six months in the same shop, and present at the time of the visit were eligible for inclusion, while those unwilling to participate were excluded. Although no formal power calculation was performed, the recruited sample of 65 barbers provides preliminary, descriptive insights into local knowledge, attitudes, and practices. As this was a convenience sample, its limited representativeness is acknowledged as a methodological limitation that affects generalizability.

Data collection

Data were collected over a five-month period from July 2024 to November 2024. A pre-designed, structured questionnaire was administered on-site, supplemented by direct observation of barbers during routine hair-cutting and shaving procedures. Each observation session lasted approximately 10-15 minutes per participant. Observations were conducted by trained members of the research team, who used a standardized checklist to evaluate shop hygiene, ventilation, sterilization facilities, razor-changing behavior, antiseptic availability, waste disposal methods, and handwashing facilities.

The observation process was discreet to minimize the Hawthorne effect; however, the possibility of behavioral changes due to the presence of the observer is acknowledged. The questionnaire collected information regarding knowledge of hepatitis B, hepatitis C, and HIV; attitudes toward hygiene and disease prevention; routine practices; and shop hygiene conditions.

The questionnaire underwent expert review (three public health specialists) to ensure face and content validity. A short pilot test on five barbers was performed to refine clarity and field applicability. Data collectors received prior training in communication skills and standardized data collection procedures.

Data analysis

Collected data were coded and analyzed using Statistical Product and Service Solutions (SPSS, version 27; IBM SPSS Statistics for Windows, Armonk, NY). Descriptive statistics (frequencies, percentages, means, and standard deviations) were computed for all variables. Knowledge, attitude, and practice (KAP) scores were calculated by summing correct responses within each domain. Participants scoring ≥70% in each domain were categorized as having adequate knowledge, positive attitude, or good practice. Associations between demographic variables and KAP levels were assessed using chi-square tests or Fisher’s exact test where appropriate. A p-value of <0.05 was considered statistically significant.

Ethical considerations

Ethical clearance for the study was obtained from the Institutional Review Board (Approval No: Ref. No RC-EA-2024/138). Verbal consent was obtained from all participants, and confidentiality was ensured through anonymous identifiers. Participants were informed of their right to withdraw at any stage without any consequences.

## Results

A total of 65 barbers from Abbottabad city participated in this study. Data were collected using a structured questionnaire and supplemented with direct shop observations. Of the participants, 32 (49%) were shop owners, and 33 (51%) were hired workers. In terms of age distribution, 41.6% were between 16 and 25 years, 40% between 26 and 40 years, and 18.8% were older than 40 years. Educational status showed that 28% had no formal education, 45% were under-matriculate, and 27% had achieved matriculation or higher qualifications.

Most barbers demonstrated a generally positive attitude toward infection prevention; however, notable gaps remained. While many acknowledged the importance of safe practices, a substantial proportion still lacked clarity about the specific diseases associated with poor hygiene. As shown in Table 1, 40% of participants were unable to identify any health risks related to barbering (“Don’t know”), whereas another 40% correctly mentioned hepatitis B/C and HIV/AIDS. A smaller proportion (16.9%) recognized multiple risks, such as skin infections, hepatitis, and HIV/AIDS. This distribution indicates that, although targeted awareness of major infectious diseases was present among many barbers, a broader understanding of the health risks associated with their profession was still limited.

**Table 1:** Demonstrating diseases perceived by barbers to be transmissible through their profession (n=65).

Response	Frequency	Percentage (%)
Don’t know	26	40.0
Hepatitis B/C, HIV/AIDS	26	40.0
Skin diseases, Hepatitis, HIV/AIDS	11	16.9
Cough & Allergy	2	3.1

Sources of information regarding disease transmission varied: 19% of barbers reported learning from television, 20% from health care professionals, 17% from friends, and 31% did not provide an answer.

**Figure 1:**
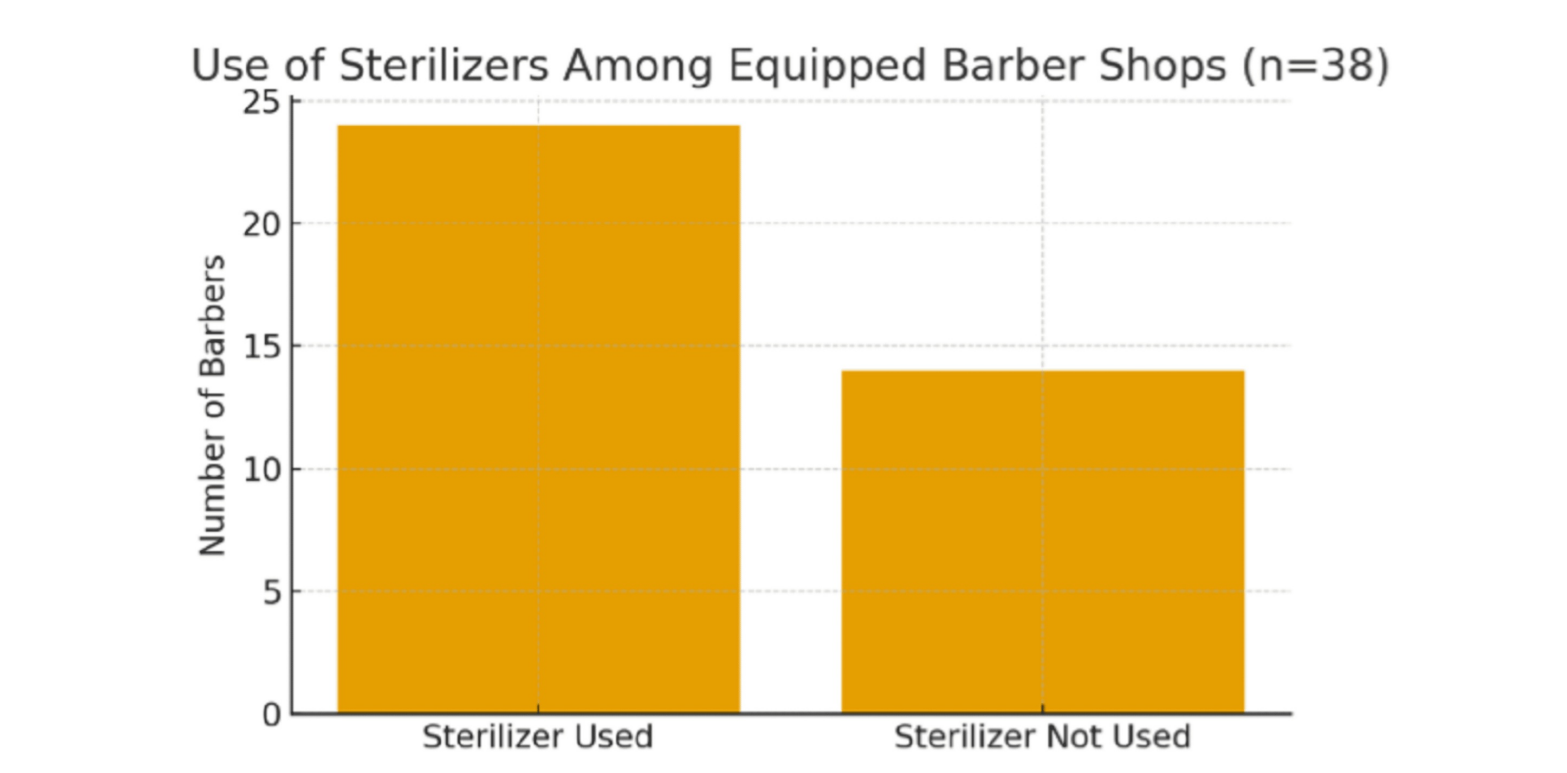
Demonstrating consistent use of sterilizers among barbers with sterilizers available (n=38).

**Figure 2:**
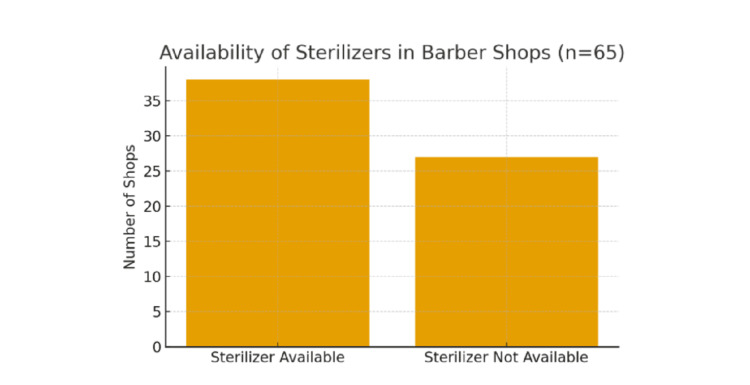
Availability of sterilizers in barber shops (n=65).

Observational assessment of barbers’ shops revealed considerable variability in hygienic and sterilization practices. As shown in Figure 1, sterilizers were available in 38 out of 65 shops (58.5%), although only 24 barbers (63% of those with sterilizers) reported using them consistently. Overall shop cleanliness was maintained in 47% of establishments, while adequate ventilation - through cross-ventilation or exhaust fans - was observed in 68% of shops. Instrument hygiene appeared satisfactory, with 78% of barbers having visibly clean tools at the time of inspection. Razor-related practices were notably better; Figure 2 illustrates that 95% of barbers reported using a new razor blade for each customer, and 94% kept antiseptic agents such as chloroxylenol liquid for routine disinfection and wound care. Overall, although basic hygiene measures are widely adopted, sterilization practices remain inconsistent, despite the availability of sterilizing equipment in many shops.

The overall hygiene of barber shops was assessed by the observers during visits. Among the 65 shops evaluated, 31 (48%) were rated as Good, 22 (34%) as Fair, and 12 (18%) as Poor. Shops rated as good maintained clean floors, disinfected instruments, used sterilizers, new razor blades for each customer, and had antiseptics available. Shops rated as fair partially followed these practices, while those rated poor lacked most hygiene measures. This assessment highlights the variability in hygiene standards among barbers in Abbottabad city.

## Discussion

This study was conducted to analyze the current situation of awareness and practices adopted by barbers according to their level of knowledge. In barbershops, clients may be exposed not only to contaminated instruments but also to various chemical and thermal hazards, as well as infectious diseases such as ringworm, pediculosis, infestations of head lice, and other scalp infections.

Through our study, we found that the majority of barbers (75.4%, n=49) were aware that diseases could be transmitted through their profession. Comparatively, a study conducted in Busia District, Uganda, revealed that 70% of respondents had some knowledge about health hazards associated with their profession [13].

A study conducted in Malayer City, Iran, demonstrated even higher awareness, where 86.66% of participants reported correct awareness of regulations, 92.28% expressed a positive attitude toward such regulations, and 86.38% showed appropriate health practices [14]. The lower specific disease knowledge observed in Abbottabad (40% naming hepatitis/HIV) compared with studies such as Ethiopia (51%) may reflect several contextual factors rather than a simple deficit in awareness. Possible explanations include differences in local health education infrastructure and outreach, the presence or absence of formal regulatory enforcement and training for barbers, and socioeconomic constraints that limit access to training and effective disinfectants. Other contributing factors may be literacy levels among barbers, differences in media and public health messaging targeted at informal workers, variability in access to healthcare professionals for advice, and practical barriers such as intermittent electricity or the cost of consumables that reduce the consistent use of sterilization equipment. These contextual differences likely explain regional variation and underscore the need for locally tailored interventions - combining education, affordable supplies, and regulatory/occupational support - to improve specific knowledge and practice in Abbottabad.

When further inquired about the exact nature of hazards, it became evident that detailed knowledge was limited. In our study, 40% (n=19) of barbers reported that they did not know the names of diseases transmissible through their profession. Although 40% of barbers in Abbottabad correctly identified hepatitis and AIDS as potential occupational risks, corresponding to 20 of the 65 barbers, this percentage is lower than that reported in the Ethiopian KAP study [8], where 51% of respondents were aware of the possible transmission of HIV through contaminated equipment. These variations likely reflect differences in health education systems, regulatory oversight, and access to structured awareness programs. Ethiopia has benefited from long-standing community-based HIV campaigns and non-governmental organization (NGO) involvement, which may explain the comparatively higher awareness levels. In contrast, the study from Nagpur City, India [15], reported that 81% of roadside barbers were unaware of HIV transmission modes, particularly via blades. This extremely low awareness level highlights the impact of informal work settings, low literacy, and the near absence of regulatory enforcement. Abbottabad appears to fall in the middle of this spectrum-barbers possess general awareness through mass media exposure and peer practices, but the absence of mandatory training, variable literacy, limited involvement of health authorities, and socioeconomic constraints contribute to gaps in disease-specific knowledge. Thus, differences across settings likely arise from broader structural factors rather than knowledge alone.

To explain these contrasts, differences in health communication systems must be considered. The higher proportion of barbers in our study who could not identify an information source (31%) and the lower reliance on television (19%) compared with Egypt [16] may reflect limited targeted awareness campaigns and lower engagement of media with informal-sector workers in Abbottabad. Conversely, the Egyptian study [16] showing greater reliance on television (40%) and friends/relatives (46.1%) suggests broader media penetration and stronger social network-based information flow. The higher proportion of barbers in our study reporting healthcare professionals as a source (20%) compared to Egypt (5.2%) may also indicate differences in how health systems interact with barbers in routine practice. These contextual factors likely explain the differing patterns of information sources observed between the two settings.

Knowledge application in practice is a critical component of disease prevention. In our study, the most common preventive measure was the use of chloroxylenol liquid, reported by 78.4% of barbers. In Islamabad [17], a study found higher compliance with preventive measures: 77.5% washed hands before every customer, 93.5% cleaned instruments before every use, 97.5% used new blades, and 60.5% managed cuts with potash alum. Similarly, in Cameroon [18], instruments were sterilized in only 10% of cases and disinfected in 72.5%, while 17.5% of sessions involved no decontamination at all. In contrast, a study from Egypt’s Gharbia district [15] showed that 55.8% of barbers used antiseptics, including Savlon (49.1%), alcohol (37.9%), povidone-iodine (8.7%), and hydrogen peroxide (10.6%), with chloroxylenol liquid being less commonly used (5%).

These findings suggest that, while Abbottabad barbers demonstrate some preventive practices, socioeconomic constraints and limited awareness hinder their adoption of diverse and more effective disinfectants. Observational findings in our study further reinforced the importance of hygienic practices. Almost half of the barbers (47%) maintained clean working environments, about 68% had adequate ventilation, and 78% kept instruments visibly clean.

This study has several important limitations. The use of convenience sampling restricts the generalizability of the findings, as only barbers who were available and willing to participate during field visits were included. Although the questionnaire was expert-reviewed and pilot-tested, its internal consistency was not formally assessed, which may affect reliability - especially for attitude-related items. The observational assessment was conducted during a single visit to each shop, introducing the possibility of the Hawthorne effect. Additionally, some factors discussed in the interpretation, such as socioeconomic status or perceived occupational risk, were not directly measured. As a cross-sectional study, causal relationships cannot be established; thus, the findings should be viewed as preliminary and hypothesis-generating.

Overall, this study demonstrates that, although awareness among barbers in Abbottabad is moderate, many preventive practices are already in place, whether intentionally or unintentionally. However, gaps remain in knowledge regarding disease transmission and in the adoption of modern disinfection methods. To strengthen practical relevance, we recommend that district health authorities and local municipal bodies lead structured awareness efforts for barbers. A simple model would involve monthly outreach visits by health workers to provide brief demonstrations on disease transmission, blade hygiene, and sterilizer use. Regulatory oversight could be improved by introducing a basic Hygiene Compliance Card linked to annual shop license renewal. These targeted, low-cost interventions offer clear mechanisms to improve hygiene practices in barber settings.

## Conclusions

Barbers in Abbottabad demonstrated moderate knowledge of general health hazards, though a detailed understanding of specific diseases and infection-control procedures remained limited. While most barbers practiced basic hygiene measures - such as maintaining shop cleanliness, using new blades, and applying disinfectants - their practices did not consistently meet recommended standards, largely reflecting resource and training gaps. The findings represent only the urban barber population, as rural barbers and those performing minor surgical procedures were excluded from the study design, which limits the generalizability of the results. Strengthening targeted hygiene training and improving access to affordable disinfection resources may help enhance safe grooming practices in this setting.

## Appendices

Annexure 1

Questionnaire for Barbers on Knowledge, Attitudes, and Practices Regarding Hygiene and Communicable Diseases

**Table 2:** (Annexure 1) Questionnaire for barbers on knowledge, attitudes, and practices regarding hygiene and communicable diseases.

Section	Question	Response Options /Notes
A. Demographic Information	1. Age	___ years
	2. Education level	☐ No formal education ☐ Primary ☐ Middle ☐ Matric ☐ Intermediate or above
	3. Designation	☐ Shop Owner ☐ Working Barber
	4. Duration of work experience	___ years
	5. Number of customers per day	___
	6. Type of shop	☐ Roadside ☐ Commercial / Urban ☐ Rural / Locality-based
B. Knowledge Regarding Communicable Diseases	7. Can diseases be transmitted through barbering instruments?	☐ Yes ☐ No ☐ Don’t know
	8. If yes, which diseases can be transmitted through barbering?	☐ Hepatitis B ☐ Hepatitis C ☐ HIV/AIDS ☐ Skin infections ☐ Others (specify) __________
	9. Do you know how these infections are spread?	☐ Yes ☐ No
	10. What is the main cause of spread?	☐ Sharing blades ☐ Contaminated razors ☐ Lack of sterilization ☐ Others __________
	11. Source of information about these diseases	☐ Television ☐ Health professionals ☐ Friends ☐ Social media ☐ None ☐ Others __________
C. Practices Related to Hygiene and Prevention	12. Do you use new razor blades for each customer?	☐ Always ☐ Sometimes ☐ Never
	13. How do you clean instruments after each use?	☐ Water only ☐ Water + Dettol ☐ Alcohol/spirit ☐ Flame sterilization ☐ UV/chemical sterilizer ☐ Don’t clean
	14. Do you have a sterilizer in your shop?	☐ Yes ☐ No
	15. If yes, do you regularly use the sterilizer?	☐ Yes ☐ No
	16. Which antiseptics are available in your shop?	☐ Dettol ☐ Spirit ☐ Savlon ☐ None ☐ Others __________
	17. Do you wash hands before every customer?	☐ Always ☐ Sometimes ☐ Never
	18. How do you manage cuts or wounds during shaving?	☐ Use antiseptic ☐ Potash alum ☐ Tissue only ☐ No action
	19. Do you wash aprons/towels regularly?	☐ Daily ☐ Weekly ☐ Monthly ☐ Rarely
	20. Do you dispose of used blades safely?	☐ Yes (Sharps box/container) ☐ No (general waste)
D. Observational Checklist (to be filled by researcher)	21. Shop cleanliness	☐ Clean ☐ Moderately clean ☐ Dirty
	22. Ventilation of shop	☐ Adequate ☐ Poor
	23. Cleanliness of instruments	☐ Clean ☐ Dirty
	24. Availability of sterilizer	☐ Yes ☐ No
	25. Actual use of sterilizer	☐ Yes ☐ No
	26. Use of new razor for each customer (observed)	☐ Yes ☐ No
	27. Availability of antiseptics (Dettol/spirit)	☐ Yes ☐ No
	28. Waste disposal method observed	☐ Safe (separate container) ☐ Unsafe (open disposal)
	29. Presence of handwashing facility	☐ Yes ☐ No
	30. Overall hygiene rating (observer’s assessment)	☐ Good ☐ Fair ☐ Poor
